# Arbuscular Mycorrhiza Symbiosis Enhances Water Status and Soil-Plant Hydraulic Conductance Under Drought

**DOI:** 10.3389/fpls.2021.722954

**Published:** 2021-10-14

**Authors:** Mohanned Abdalla, Mutez Ali Ahmed

**Affiliations:** ^1^Chair of Soil Physics, Bayreuth Center of Ecology and Environmental Research (BayCEER), University of Bayreuth, Bayreuth, Germany; ^2^Department of Horticulture, Faculty of Agriculture, University of Khartoum, Khartoum North, Sudan

**Keywords:** Soil drying, AMF, rhizosphere, root water uptake, biostimulant, plant microbiome, abiotic stress

## Abstract

Recent studies have identified soil drying as a dominant driver of transpiration reduction at the global scale. Although Arbuscular Mycorrhiza Fungi (AMF) are assumed to play a pivotal role in plant response to soil drying, studies investigating the impact of AMF on plant water status and soil-plant hydraulic conductance are lacking. Thus, the main objective of this study was to investigate the influence of AMF on soil-plant conductance and plant water status of tomato under drought. We hypothesized that AMF limit the drop in matric potential across the rhizosphere, especially in drying soil. The underlying mechanism is that AMF extend the effective root radius and hence reduce the water fluxes at the root-soil interface. The follow-up hypothesis is that AMF enhance soil-plant hydraulic conductance and plant water status during soil drying. To test these hypotheses, we measured the relation between transpiration rate, soil and leaf water potential of tomato with reduced mycorrhiza colonization (RMC) and the corresponding wild type (WT). We inoculated the soil of the WT with *Rhizophagus irregularis* spores to potentially upsurge symbiosis initiation. During soil drying, leaf water potential of the WT did not drop below −0.8MPa during the first 6days after withholding irrigation, while leaf water potential of RMC dropped below −1MPa already after 4days. Furthermore, AMF enhanced the soil-plant hydraulic conductance of the WT during soil drying. In contrast, soil-plant hydraulic conductance of the RMC declined more abruptly as soil dried. We conclude that AMF maintained the hydraulic continuity between root and soil in drying soils, hereby reducing the drop in matric potential at the root-soil interface and enhancing soil-plant hydraulic conductance of tomato under edaphic stress. Future studies will investigate the role of AMF on soil-plant hydraulic conductance and plant water status among diverse plant species growing in contrasting soil textures.

## Introduction

Water scarcity in soil and atmosphere escalates stress on vegetation and threatens future agricultural production and forest survival, especially in the face of climate change (Madadgar et al., 2017; Brodribb et al., 2020). Recent studies have identified soil drying as a primary cause of transpiration reduction globally, which is a greater stress factor than vapor pressure deficit (VPD; Liu et al., 2020). Thus, detailed knowledge of water flow processes, particularly belowground, is required to fully understand and predict plant behavior under drought episodes and future climate conditions.

Water flow across the soil-plant-atmosphere continuum is driven by gradients in water potential between the atmosphere and soil. Water evaporation at the leaf surface (i.e., due to the increase in the vapor pressure deficit) creates a tension that propagates down to the roots and the soil. The leaf water potential (*ψ*_leaf_) depends on both water potential in the soil (*ψ*_soil_) and the hydraulic conductivities of the different elements (soil, root-soil interface, root, xylem, and leaf) composing the soil-plant continuum. Sperry and Love (2015) used a hydraulic model of water flow to propose that stomata regulation allows plants not to exceed the water supply function determined by soil-plant hydraulics. In other words, downregulation of stomata in dry conditions avoids an excessive decline in leaf water potential before approaching a critical transpiration rate. This hypothesis implies that the leaf water potentials at which stomata close depend also on belowground hydraulic properties (root, soil, and their interface). Despite their importance, studies investigating the impact of belowground traits on plant water status and soil-plant hydraulic conductance remain limited.

In wet soils, the hydraulic conductivity of soil is much higher than that of roots and hence, water flow is mainly controlled by root hydraulic conductivity (Draye et al., 2010). However, as soil dries, its conductivity drops by a few orders of magnitude, limiting the water flow toward the root surface (Passioura, 1980; Draye et al., 2010). Indeed, Carminati and Javaux (2020) combined a soil-plant hydraulic model with meta-analysis to elucidate that the loss in soil conductivity, especially at the root-soil interface, controls stomatal response during water deficit. Similarly, we have recently showed that, in tomato, the decline in soil-root hydraulic conductance was the main driver of stomatal closure (Abdalla et al., 2021). Rodriguez-Dominguez and Brodribb (2020) used a novel rehydration technique to demonstrate that the loss in hydraulic conductivity at the root-soil interface occurred in parallel with stomatal closure. In a follow-up study, Bourbia et al. (2021) showed that a decline in root hydraulic conductivity was concomitant with a stomatal closure in both herbaceous and woody species. Plants developed various strategies to deal with the drop of conductivity at the root-soil interface (Carminati et al., 2016; Ahmed et al., 2018a). For instance, in barley, root hairs were not only documented to soften the gradients in matric potential at the root-soil interface (Carminati et al., 2017) but also enhance plant water status and yield during water deficit (Marin et al., 2020). Another example is mucilage, a gel exuded at the root tip, which was shown to facilitate water uptake in drying soils (Ahmed et al., 2014). Furthermore, root-microbiome interactions (e.g., Arbuscular mycorrhiza fungi) provide fitness advantages to the host plant to mitigate water stress conditions [reviewed in Trivedi et al. (2020)].

Arbuscular mycorrhiza fungi (AMF) symbiosis, which occurs naturally between fungal and most plant species, is documented to play a positive role in plant water relations, especially under water deficit (Augé, 2001; Augé et al., 2015; Ouledali et al., 2018). For instance, Bitterlich et al. (2018b) showed that, in tomato, AMF facilitated higher transpiration rates in dry soils. Similarly, Chitarra et al. (2016) demonstrated that AMF enhanced tomato performance under water stress. The authors showed that AMF improved plant biomass and water use efficiency (Chitarra et al., 2016). In maize, Quiroga et al. (2019) reported that AMF symbiosis enabled higher stomatal conductance under soil water deficit. Furthermore, it was also suggested that AMF increase root hydraulic conductivity (Aroca et al., 2007; Quiroga et al., 2019) and alter soil hydraulic properties (Bitterlich et al., 2018a; Pauwels et al., 2020). However, the impact of AMF on soil-plant hydraulic conductance, especially in drying soil, remains unknown. Note that this would be crucial to improve our current understanding of plant response to drought (Carminati et al., 2020; Hayat et al., 2020; Rodriguez-Dominguez and Brodribb, 2020; Abdalla et al., 2021). Thus, there is an urgent need to investigate the influence of AMF on soil-plant hydraulic conductance during soil drying.

**Figure 1:**
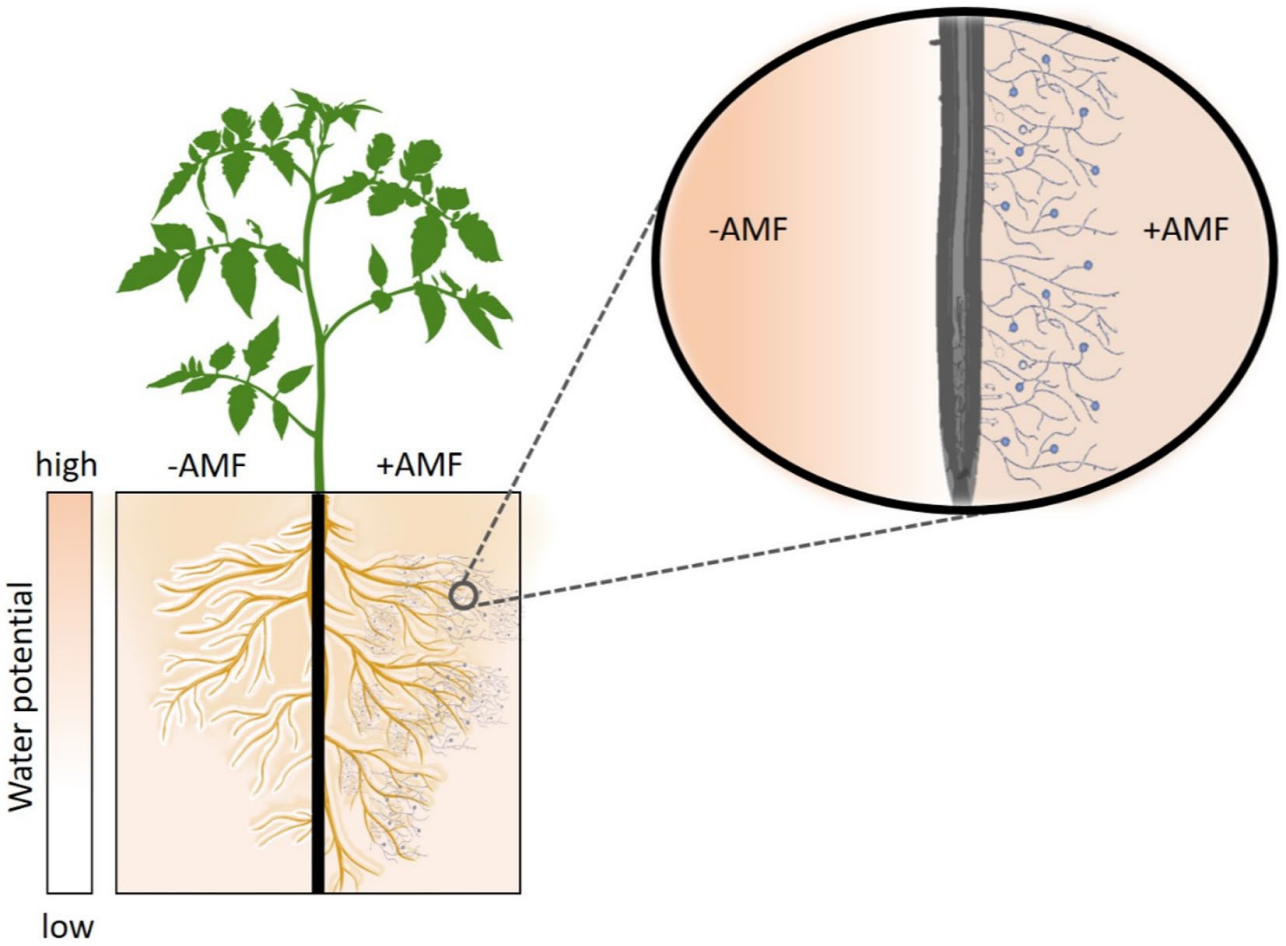
Hypothetical role of Arbuscular Mycorrhiza Fungi (AMF) in enhancing plant water status and soil-plant hydraulic conductance. During soil drying, AMF increase the root-soil contact and extend the effective root radius hereby reducing the water fluxes at the root-soil interface and softening the drop in matric potential across the rhizosphere. The follow-up hypothesis is that AMF enhance soil-plant hydraulic conductance and plant water status during soil drying. Plants without AMF symbiosis (-AMF) require larger gradients in matric potential around their roots to sustain similar transpiration rates.

We hypothesize that AMF increase the root-soil contact and hence the effective root radius, especially in dry soil. This would reduce the flow velocity at the root surface and soften the drop in matric potential at the root-soil interface (Figure 1). This, in turn, would facilitate higher (less negative) leaf water potential and enhance soil-plant hydraulic conductance during soil drying.

We tested this hypothesis in tomato plants inoculated with *Rhizophagus irregularis* spores. We utilized mutant variety with highly reduced AMF symbiosis and the corresponding wild type. We measured transpiration rate, soil water content, water potentials in soil and leaf during soil drying. We used the relation between transpiration rate and leaf water potential to infer the hydraulic conductance of the soil-plant system for both genotypes during soil drying.

## Materials and Methods

### Plant Preparation

We used two tomato genotypes (*Solanum lycopersicum* L.): a mutant with highly reduced AMF symbiosis (RMC) and its wild-type counterpart (WT; Barker et al., 1998). Growth was shown to be very similar in both genotypes, suggesting no pleiotropic effects of the mutation (Cavagnaro et al., 2004). Seeds were sterilized in 30% H_2_O_2_ for 90s and thereafter washed and germinated on Petri dishes. The seeds were then planted in PVC columns of 30cm in height and 9cm in diameter. The columns had small five holes on the side, which were used for soil water content measurements during the experiment. The columns were filled with sandy soil through a 1-mm sieve. The hydraulic properties and fertilization of the soil are reported in Vetterlein et al. (2021) and in supplementary information (Supplementary Figure S1). To potentially upsurge AMF colonization of the WT, the soil was inoculated with commercial *R. irregularis* spores (BIOFA AG, Münstingen, Germany) in a ratio of 50 spore kg^−1^.

### Growth Conditions

Twenty plants (10 per genotype) were placed in a climate-controlled chamber with a day/night temperature of 29/19°C, a day/night relative humidity of 51/79%, 14h of photoperiod, and light intensity of 1,000μmolm^−2^ s^−1^. Plants were randomized inside the chamber. The soil surface was covered with polyolefin to prevent evaporation. We measured shoot fresh weight at the end of the experiment.

### Transpiration Rate

Plants were placed into wireless balances that automatically recorded the changes in weight every 10mins. Transpiration rate was obtained gravimetrically by calculating the difference in weight over time. We extracted the transpiration rate for predawn (no light and low VPD) and midday. Plants were irrigated daily until the start of measurements.

### Leaf Water Potential Measurements

After withholding irrigation, leaf water potential was measured on daily basis at midday. A leaflet was covered with a plastic bag and lined with aluminum foil for at least 20mins before measurement. Covered leaves were cut and placed inside a Scholander-type pressure chamber (MODEL 3115, Soil Moisture Equipment Corp, Santa Barbara, CA, Unites States) to obtain stem water potential, which was used as a proxy for leaf water potential (One leaflet was measured per plant).

### Soil Dryness Assessment

Soil water content (*θ*) was measured daily using time-domain refractometer that encompasses two rods (spacing: 0.5cm; length: 6cm) connected to a data logger (E-Test, Lublin, Poland). Soil water potential was computed from the soil water content using the soil water retention curve (Supplementary Figure S1).

### Soil-Plant Hydraulic Conductance

During soil drying, soil-plant hydraulic conductances of RMC and WT were obtained using Equation (1) as follow:


Ksp=EΔψ (1)

where *Ksp* is soil-plant hydraulic conductance (cm^3^ s^−1^ MPa^−1^), *E* is transpiration rate (cm^3^ s^−1^), and Δ*ψ* is the difference between absolute values of leaf and soil water potentials (MPa).

### AMF Abundance Assessment

Roots were collected at the end of the experiments and stored in 60% ethanol. Root samples of both genotypes were washed with distilled water, cleared with 5% KOH, and stained in 5% ink-vinegar solution to visualize AMF colonization in roots [after Vierheilig et al. (1998)]. The percentage of colonized root length was determined by recording 150 root-intersects per sample using the light microscopy (Olympus BX40) and the attached digital camera (Olympus SC50).

### Statistical Analysis

ANOVA was used to identify significant differences in transpiration rates, leaf water potential, and soil-plant hydraulic conductance of WT and RMC. T-test was applied to evaluate the differences in root colonization between the WT and RMC mutant. MATLAB (R2019) was used to perform the statistical analysis.

## Results and Discussion

**Figure 2:**
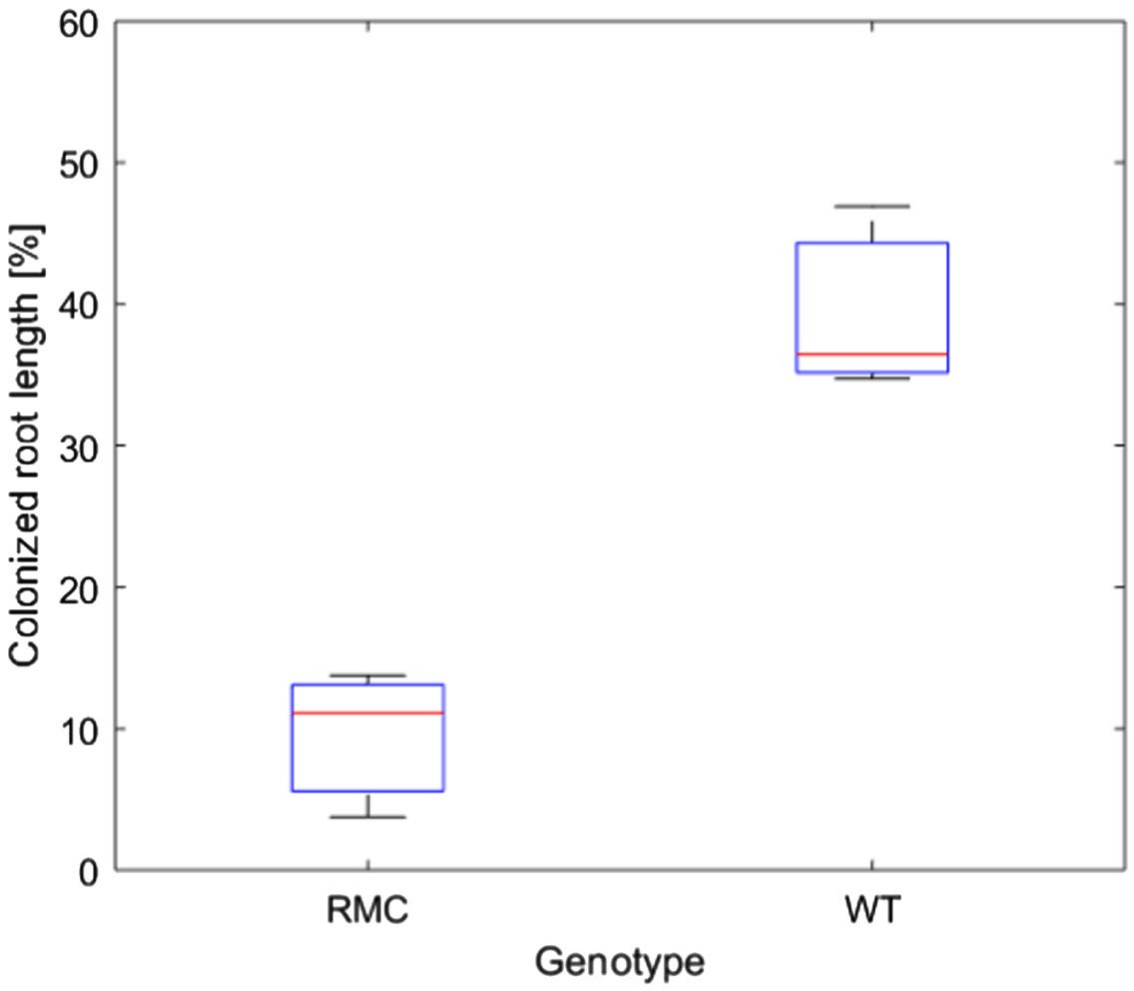
AMF abundance assessment in roots of reduced mycorrhiza colonization (RMC) and wild-type counterpart (WT). AMF colonization was four times higher in WT compare to RMC (value of *p*<0.05).

We investigated the impact of AMF on plant water status and soil-plant hydraulic conductance in two tomato genotypes, reduced mycorrhiza colonization (RMC) and its wild type counterpart (WT). Plant biomass of both genotypes was similar (Supplementary Figure S2). Shoot fresh weight was 30.2±8.2g and 28.4±7.9g for the WT and RMC, respectively (Supplementary Figure S2). The root colonization of the WT was four times higher than RMC (value of *p*<0.05; Figure 2). This finding is consistent with results of Zhou et al. (2020), who assessed AMF root colonization in same tomato genotypes and observed significantly higher AMF abundance in roots of WT compared to RMC.

**Figure 3:**
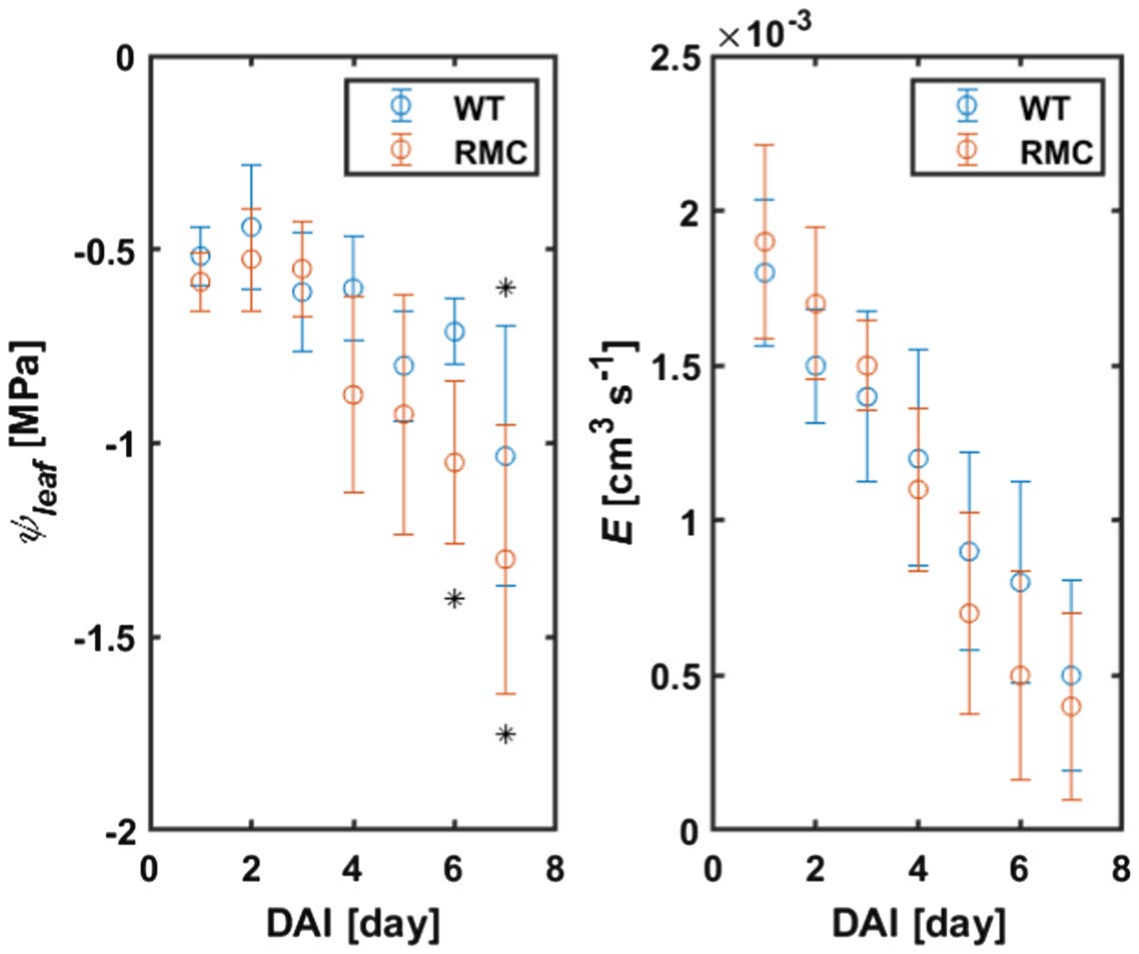
Leaf water potential (*ψ*_leaf_) and transpiration rate (*E*) of RMC and WT tomato during soil drying. *ψ*_leaf_ declined markedly in the absence of AMF, which nicely support our initial hypothesis. Asterisks denote significance decline of *ψ*_leaf_ (value of *p*<0.01), on top for WT and bottom for RMC. DAI, day after last irrigation. *n*=10.

Leaf water potential of the WT plants did not drop below −0.8MPa 6days after withholding irrigation, while leaf water potential of the RMC dropped below −1.0MPa already after 4days (value of *p*<0.01; Figure 3; Supplementary Table S1). These results are in line with previous findings in maize, soybean, and barley (Subramanian et al., 1997; Porcel, 2004; Khalvati et al., 2005). The authors showed that, under water deficit, plants with AMF colonization exhibited higher (less negative) leaf water potential compared to plants without AMF.

Transpiration declined in both treatments as a consequence of water deficit (Figure 3). During soil drying, we observed, surprisingly, no differences in transpiration rate between the two genotypes (value of *p*=0.5, Supplementary Table S2). These results are in line with the findings of Chitarra et al. (2016), who reported similar stomatal conductance of tomato inoculated with different AMF species and the control [see Figure 1B in Chitarra et al. (2016)]. Despite the absence of difference in transpiration rate, the authors compared the water use efficiency and demonstrated that AMF improved tomato performance under water deficit (Chitarra et al., 2016). Similar transpiration rate was also observed in inoculated and not inoculated common bean (Aroca et al., 2007). On the other hand, Bitterlich et al. (2018b) showed that, in tomato, AMF facilitate higher transpiration in dry soil. Similarly, Hallett et al. (2009) used the same genotypes and reported a significant increase in transpiration of the wild type compared to RMC mutant. These apparently contradicting findings on the impact of AMF on transpiration rate clearly suggest that the role of AMF on transpiration (and stomatal conductance) is soil, species, and environment specific. Hence, the impact of AMF on transpiration on some of these studies might have been masked out as a result of species×environment interactions, which is well known to impact transpiration (Vadez et al., 2013, 2021). Indeed, an improved performance of AMF treatment was shown in field experiments compare to greenhouse and climate-controlled experiments (where normally plants are grown in pots; Poorter et al., 2012; Augé et al., 2015). The fact that the two genotypes exhibited no significant difference in transpiration in the present study could be explained by the limited soil volume. Hence, plants and AMF had to share a limited amount of water (and nutrients) within the pot (Chitarra et al., 2016). Moreover, this explanation can potentially justify the drop in leaf water potential in both genotypes on the seventh day after withholding irrigation (Figure 3).

In previous studies, simultaneous measurements of transpiration rate and leaf water potential with high temporal resolution revealed that leaf water potential drops rapidly when a critical transpiration rate is reached at a given soil water potential (Carminati et al., 2017; Abdalla et al., 2021; Cai et al., 2021). In other words, at a specific transpiration rate, leaf water potential can vary based on belowground hydraulic conductance [see Cai et al. (2021)]. In this study, we observed a decoupling in the relation between transpiration and leaf water potential (Figure 3). We explain this by the fact that RMC plants require larger gradients in soil water potential at the root-soil interface to sustaining a similar transpiration rate to the WT. The underlying mechanisms is that AMF extends the root surface active in water uptake, which reduces the flow velocity and attenuate the drop in matric potential at the root surface (see Figure 1). Hence, RMC plants exhibited more negative leaf water potential to sustain a similar transpiration rate as in WT. This would explain why RMC plants displayed more negative leaf water potential while maintaining similar transpiration rate as WT plants. These results demonstrate that the relation between transpiration rate and leaf water potential is not unique and depends on belowground hydraulics. Moreover, our data show that AMF clearly affect this relation. More work would be needed to test the impact of AMF on this relation among diverse plant species, contrasting soil types, and climatic conditions.

Another possible explanation for similar transpiration rate is that AMF colonization might influence the stomatal density. Chitarra et al. (2016) demonstrated that inoculation with *Rhizophagus intraradices* induced two times stomatal density compared to un-inoculated tomato plants or inoculated with *Funneliformis mosseae*. However, a different AMF species was used in this study, namely, *R. irregularis*, and its influences on stomatal density in tomato are yet to be explored. Nevertheless, our data on leaf water potential suggest that AMF could contribute positively, allowing tomato plants to mitigate water stress conditions.

**Figure 4:**
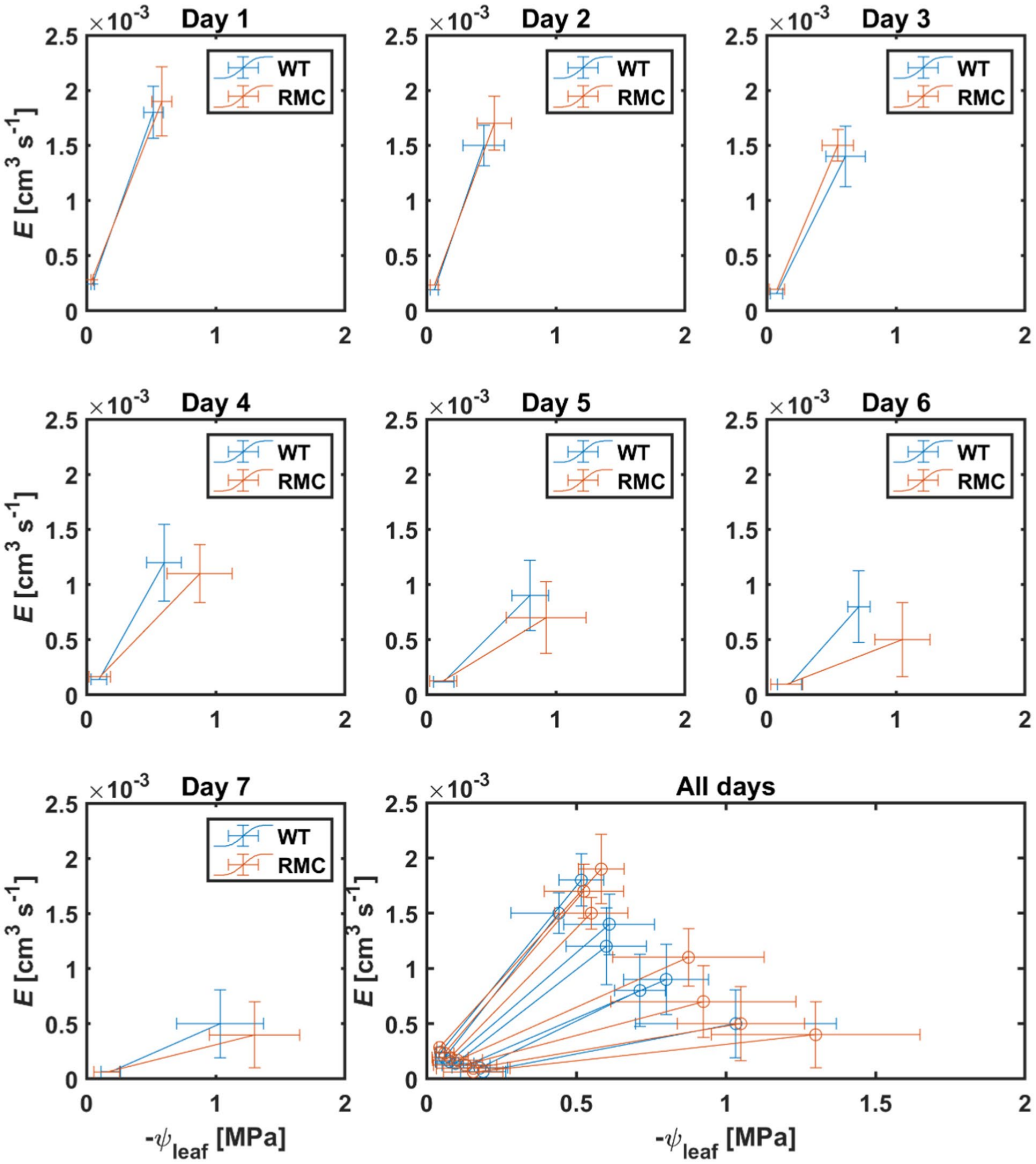
Relation between transpiration rate (*E*) and leaf water potential (*ψ*_leaf_) during soil drying. Subplots show the relation on daily basis after last irrigation. As soil progressively dried, the RMC plants showed lower *E* and more negative *ψ*_leaf_ on the same day compare to WT. *n*=10.

**Figure 5:**
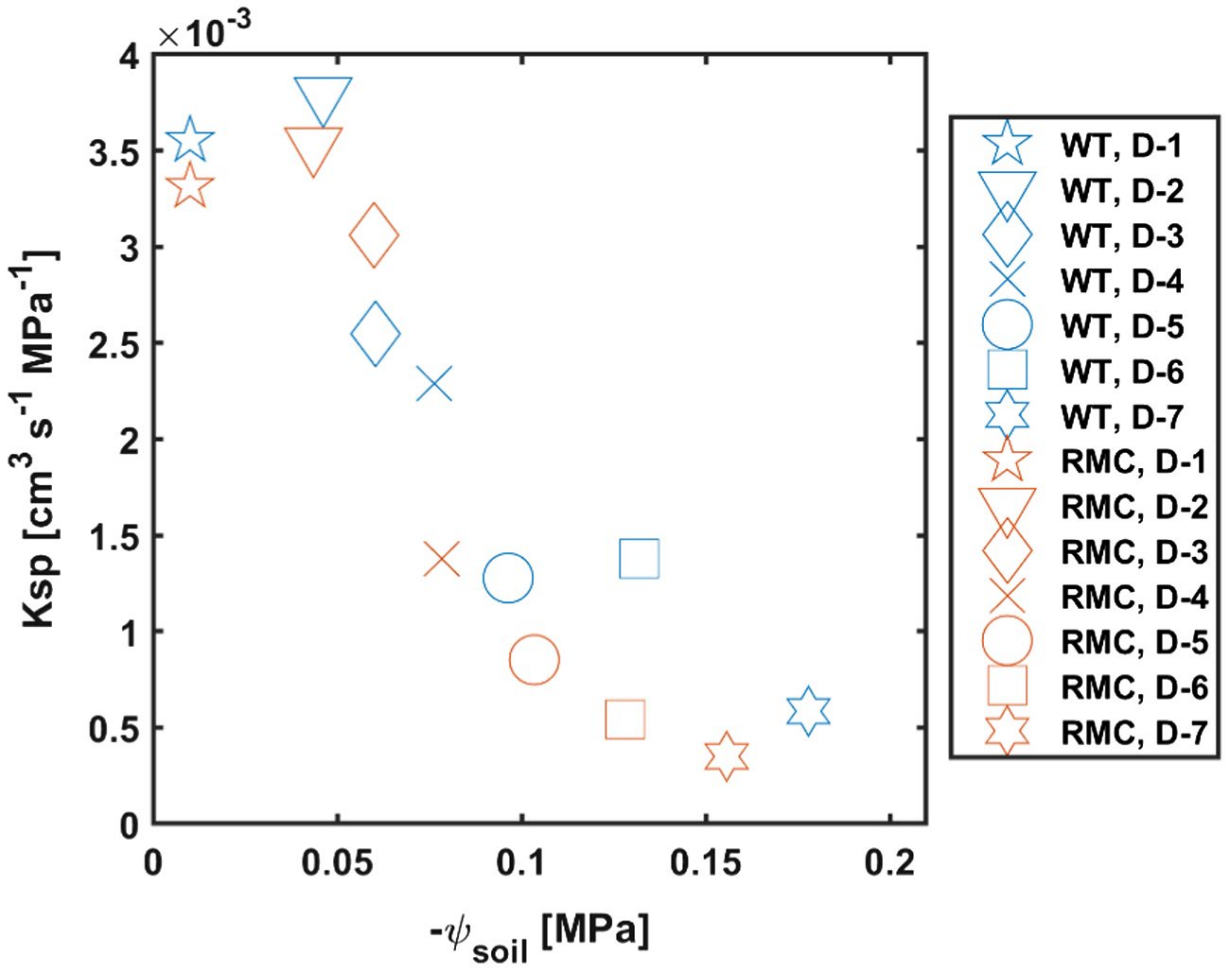
Soil-plant hydraulic conductance (*Ksp*) decreases as soil water potential (*ψ*_soil_) declines. Plants with mycorrhiza symbiosis (WT) show higher *Ksp* during soil drying (similar symbols and diverse colors) compare to RMC mutants. D, day after last irrigation. *n*=10.

During soil drying, the relation between transpiration and leaf water potential was affected by AMF colonization (Figure 4). In wet conditions, i.e., day one, both genotypes showed high transpiration and leaf water potential (Figure 4). As soil progressively dried, RMC showed relatively lower transpiration and more negative leaf water potential than the WT (Figure 4). Soil-plant hydraulic conductance (*Ksp*) was obtained from the relation between transpiration rate and leaf water potential at a given soil water potential (Figure 5). Figure 5 shows that, during soil drying, WT plants exhibited a higher *Ksp* compared to RMC (Figure 5; value of *p*=0.06; Supplementary Table S3). Note that *Ksp* is highly dependent on both transpiration rate and leaf water potential [see Equation (1)]. The marginal difference in *Ksp* is a reflection of the similar transpiration rate and the significantly different leaf water potential between the two genotypes. This finding supports our hypothesis that AMF maintain soil-root hydraulic conductance. Further, *Ksp* of RMC plants declined at less negative soil water potential compare to WT (Figure 5). The absence of AMF in the RMC plants entailed a severe reduction in leaf water potential as soil water potential declined, possibly due to loss of contact between root and soil (Carminati et al., 2009, 2013). On the other hand, AMF presence in the WT facilitated higher leaf water potential despite declining soil water potential (Figure 3). AMF could play a central role in sustaining the hydraulic continuity between root and soil, as it not only improves the unsaturated hydraulic conductivity (Bitterlich et al., 2018a; Pauwels et al., 2020), but also avoids excessive drop of soil water potential around roots.

Taken together, we have demonstrated the direct influence of AMF on soil-plant hydraulic conductance and plant water status during soil drying. WT plants exhibited higher soil-plant hydraulic conductance and leaf water potential compared to RMC plants during soil drying. We conclude that AMF extended the effective root radius hereby reducing the water fluxes at the root-soil interface and softening the drop in matric potential across the rhizosphere. This would result in an enhanced soil-plant hydraulic conductance and plant water status in drying soil. Further research is needed to directly measure the effects of AMF on water fluxes under contrasting soil textures and nutrient availabilities. The latter could be achieved using the combination of isotopes and neutron imaging (Ahmed et al., 2016, 2018b). Our data suggest that AMF could play an essential role in achieving sustainable agricultural production with greater importance in regions faced by water scarcity conditions worldwide.

## Data Availability Statement

The original contributions presented in the study are included in the article/Supplementary Material, and further inquiries can be directed to the corresponding author.

## Author Contributions

MoA and MuA designed the study and wrote the manuscript. MoA conducted the experiments and analyzed the data. All authors contributed to the article and approved the submitted version.

## Funding

The German Academic Exchange Service (DAAD) is acknowledged for funding the doctoral position of MoA. This publication was funded by the University of Bayreuth Open Access Publishing Fund.

## Conflict of Interest

The authors declare that the research was conducted in the absence of any commercial or financial relationships that could be construed as a potential conflict of interest.

## Publisher’s Note

All claims expressed in this article are solely those of the authors and do not necessarily represent those of their affiliated organizations, or those of the publisher, the editors and the reviewers. Any product that may be evaluated in this article, or claim that may be made by its manufacturer, is not guaranteed or endorsed by the publisher.
