# Supplementary material for: Arbuscular Mycorrhiza Symbiosis Enhances Water Status and Soil-Plant Hydraulic Conductance Under Drought
Source: Front Plant Sci. 2021 Oct 14;12:722954. doi: 10.3389/fpls.2021.722954 (PMC8551442; doi:10.3389/fpls.2021.722954)
Supplement: Supplementary file 1 [file Data_Sheet_1.docx]

**Arbuscular mycorrhiza symbiosis enhances water status and soil-plant hydraulic conductance under drought**

Mohanned Abdalla^1,2*^, and Mutez Ali Ahmed^1^

Chair of Soil Physics, Bayreuth Center of Ecology and Environmental Research (BayCEER), University of Bayreuth, Bayreuth, Germany.

^2^Department of Horticulture, Faculty of Agriculture, University of Khartoum, Khartoum North, Sudan.

^*^Corresponding author:

Mohanned Abdalla Email: [Mohanned.Abdalla-Ali-Abdalla@uni-bayreuth.de](mailto:Mohanned.Abdalla-Ali-Abdalla@uni-bayreuth.de)

**Supplementary information**

**Table S1.** Analysis of variance to identify significant differences in leaf water potential between the two genotypes during soil drying.

| Source | d.f. ¶ | Sum Sq. | Mean Sq. | F | Prob>F |
| --- | --- | --- | --- | --- | --- |
| AMF  DAI  AMF * DAI | 1  6  6 | 0.38514  3.18522  0.26388 | 0.38413  0.53087  0.04398 | 9.46  13.07  1.08 | **0.0033**  **<<0.0001**  0.3848 |
| Error | 52 | 5.93955 | 0.04061 |  |  |

¶ **d.f.**: degree of freedom. **Sum. Sq**: Sum of squares, **Mean Sq**: Mean Sum of Squares, **F**: F-statistic value. **DAI**: day after last irrigation.

**Table S2.** Analysis of variance to identify significant differences in transpiration rate between the two genotypes during soil drying.

| Source | d.f. ¶ | Sum Sq. | Mean Sq. | F | Prob>F |
| --- | --- | --- | --- | --- | --- |
| AMF  DAI  AMF * DAI | 1  6  6 | 3.23e-8  1.58e-5  4.45e-7 | 3.23e-8  2.65e-6  7.42e-8 | 0.39  32.1  0.9 | 0.5  **<<0.0001**  0.5016 |
| Error | 56 | 4.62e-6 | 8.24e-8 |  |  |

¶ **d.f.**: degree of freedom. **Sum. Sq**: Sum of squares, **Mean Sq**: Mean Sum of Squares, **F**: F-statistic value. **DAI**: day after last irrigation.

**Table S3.** Analysis of variance to identify significant differences in soil-plant hydraulic conductance between the two genotypes during soil drying.

| Source | d.f. ¶ | Sum Sq. | Mean Sq. | F | Prob>F |
| --- | --- | --- | --- | --- | --- |
| AMF  Soil Water Potential | 1  6 | 0.1e-4  1.1e-4 | 5.45283e-06  1.7829e-05 | 3.61  11.82 | **0.0623**  **<<0.0001** |
| Error | 58 | 0.9e-4 | 1.50884e-06 |  |  |

¶ **d.f.**: degree of freedom. **Sum. Sq**: Sum of squares, **Mean Sq**: Mean Sum of Squares, **F**: F-statistic value.


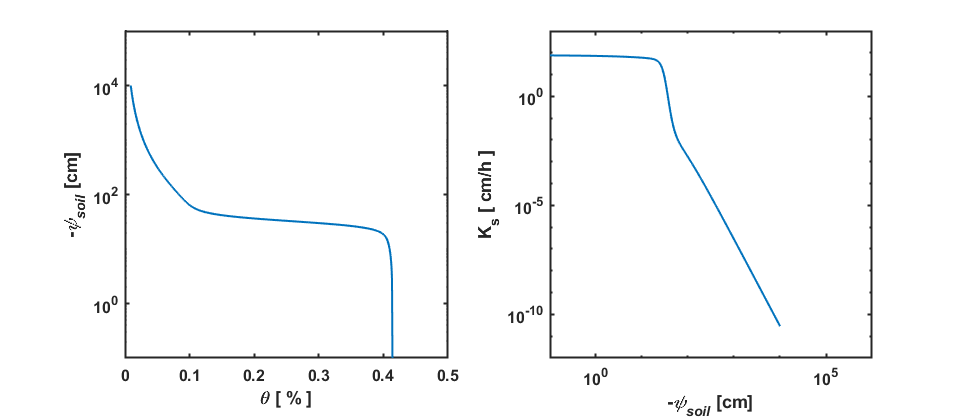


**Fig. S1:** (**a**) Soil water retention curve, and (**b**) soil hydraulic conductivity curve of sandy soil used in this experiment.


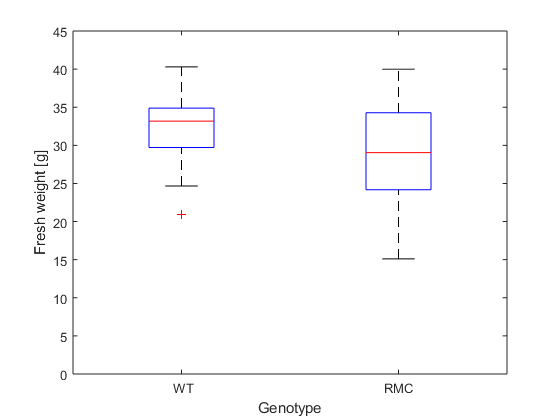


**Fig. S2:** Fresh weight of wild type (WT) and mutant (RMC) were similar. Weights were measured at the end of the experiments.


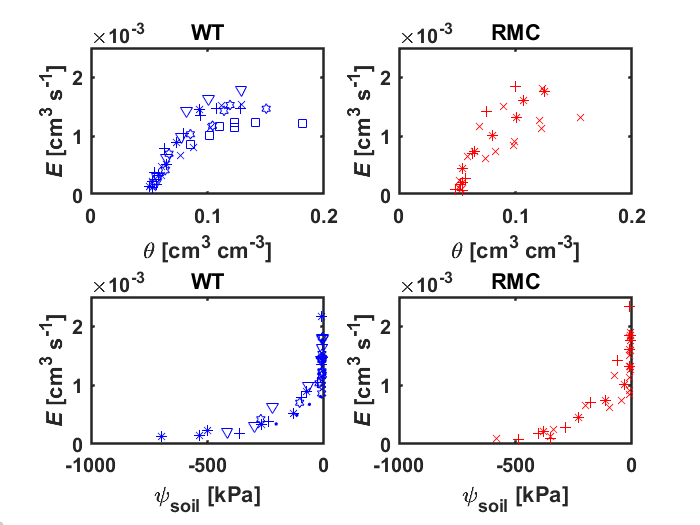


**Fig. S3:** Transpiration rate (*E*) as function of soil water content (*θ*) and soil water potential (*ψ_soil_*) in reduced mycorrhiza colonization (RMC; red) and wild type (WT; blue) plants. *E* declined gradually as *θ* decreased, while it cannot be sustained as soon as *ψ_soil_* starts to decrease. *n* = 10. Different symbols for individuals.
